# A Semisynthetic Oligomannuronic Acid-Based Glycoconjugate Vaccine against *Pseudomonas aeruginosa*

**DOI:** 10.1021/acscentsci.4c00387

**Published:** 2024-07-10

**Authors:** Yiyue Zhang, Xiaotong Wang, Youling Liang, Liangliang Zhang, Jiahao Fan, You Yang

**Affiliations:** † Shanghai Frontiers Science Center of Optogenetic Techniques for Cell Metabolism, Shanghai Key Laboratory of New Drug Design, School of Pharmacy, East China University of Science and Technology, 130 Meilong Road, Shanghai 200237, China; ‡ Engineering Research Center of Pharmaceutical Process Chemistry, Ministry of Education, East China University of Science and Technology, 130 Meilong Road, Shanghai 200237, China

## Abstract

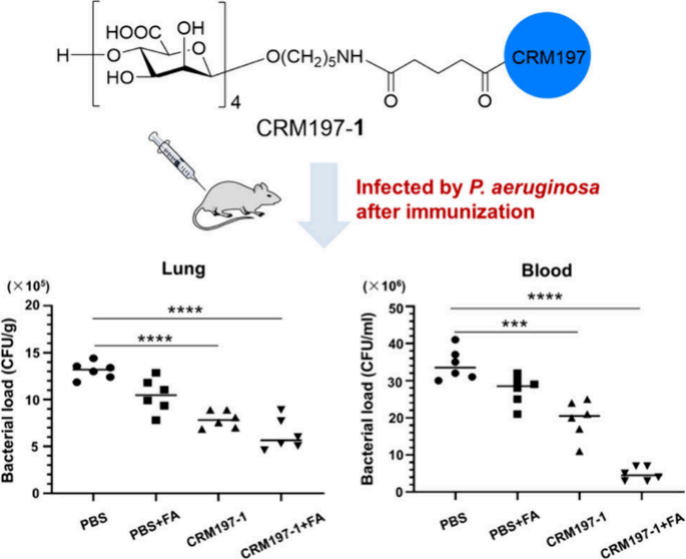

*Pseudomonas aeruginosa* is one of the leading causes of nosocomial infections and has become increasingly resistant to multiple antibiotics. However, development of novel classes of antibacterial agents against multidrug-resistant *P. aeruginosa* is extremely difficult. Herein we develop a semisynthetic oligomannuronic acid-based glycoconjugate vaccine that confers broad protection against infections of both mucoid and nonmucoid strains of *P. aeruginosa*. The well-defined glycoconjugate vaccine formulated with Freund’s adjuvant (FA) employing a highly conserved antigen elicited a strong and specific immune response and protected mice against both mucoid and nonmucoid strains of *P. aeruginosa*. The resulting antibodies recognized different strains of *P. aeruginosa* and mediated the opsonic killing of the bacteria at varied levels depending on the amount of alginate expressed on the surface of the strains. Vaccination with the glycoconjugate vaccine plus FA significantly promoted the pulmonary and blood clearance of the mucoid PAC1 strain of *P. aeruginosa* and considerably improved the survival rates of mice against the nonmucoid PAO1 strain of *P. aeruginosa*. Thus, the semisynthetic glycoconjugate is a promising vaccine that may provide broad protection against both types of *P. aeruginosa*.

## Abstract 2

A semisynthetic oligomannuronic acid-based glycoconjugate vaccine was developed to provide broad protection against infections of both mucoid and nonmucoid strains of *Pseudomonas aeruginosa*.

## Introduction

*Pseudomonas aeruginosa* is an opportunistic Gram-negative bacterium, which is one of the leading causes of nosocomial infections in immunocompromised people especially with wound infections, ventilator-associated pneumonia (VAP), and cystic fibrosis (CF).^1−4^ Early colonization of the lung of CF patients by nonmucoid strains of *P. aeruginosa* gradually evolves into chronic infection of CF lung associated with mucoid strains, ultimately resulting in the formation of biofilms against antibiotic penetration.^5−7^ Due to its ability of high adaptability to changing environments, *P. aeruginosa* has become resistant to many antibiotics including β-lactams, aminoglycosides, and quinolones.^8,9^ In 2017, *P. aeruginosa* was listed as one of the top three critical priority pathogens by the World Health Organization (WHO), for which innovative treatments are urgently needed.^10^ Among the multidrug-resistant (MDR) ESKAPE (*Enterococcus faecium*, *Staphylococcus aureus*, *Klebsiella pneumoniae*, *Acinetobacter baumannii*, *P. aeruginosa*, and *Enterobacter* species) pathogens, *P. aeruginosa* is increasingly resistant to common antibiotics and poses a great threat to human health.^11^

Despite the growing demand for antimicrobial agents with new mechanism of action in clinical practice, development of novel classes of antibiotics against MDR *P. aeruginosa* is extremely difficult as indicated by the approval of very few small molecules in the past 30 years.^12,13^ As such, new nonantibiotic approaches for prevention and treatment of MDR *P. aeruginosa* are urgently needed. Vaccination is a promising alternative strategy to prevent the infection of *P. aeruginosa* in potential at-risk populations. In view of the key role of diverse virulence factors in the pathogenicity of *P. aeruginosa*, multiple antigens including lipopolysaccharide (LPS), alginate, flagellum, outer membrane proteins, and killed whole cells have been tested in clinical trials, suggesting the vast potential of employing virulence factors as vaccine candidates.^14−16^ However, currently no vaccine against *P. aeruginosa* has been licensed.

**Figure 1:**
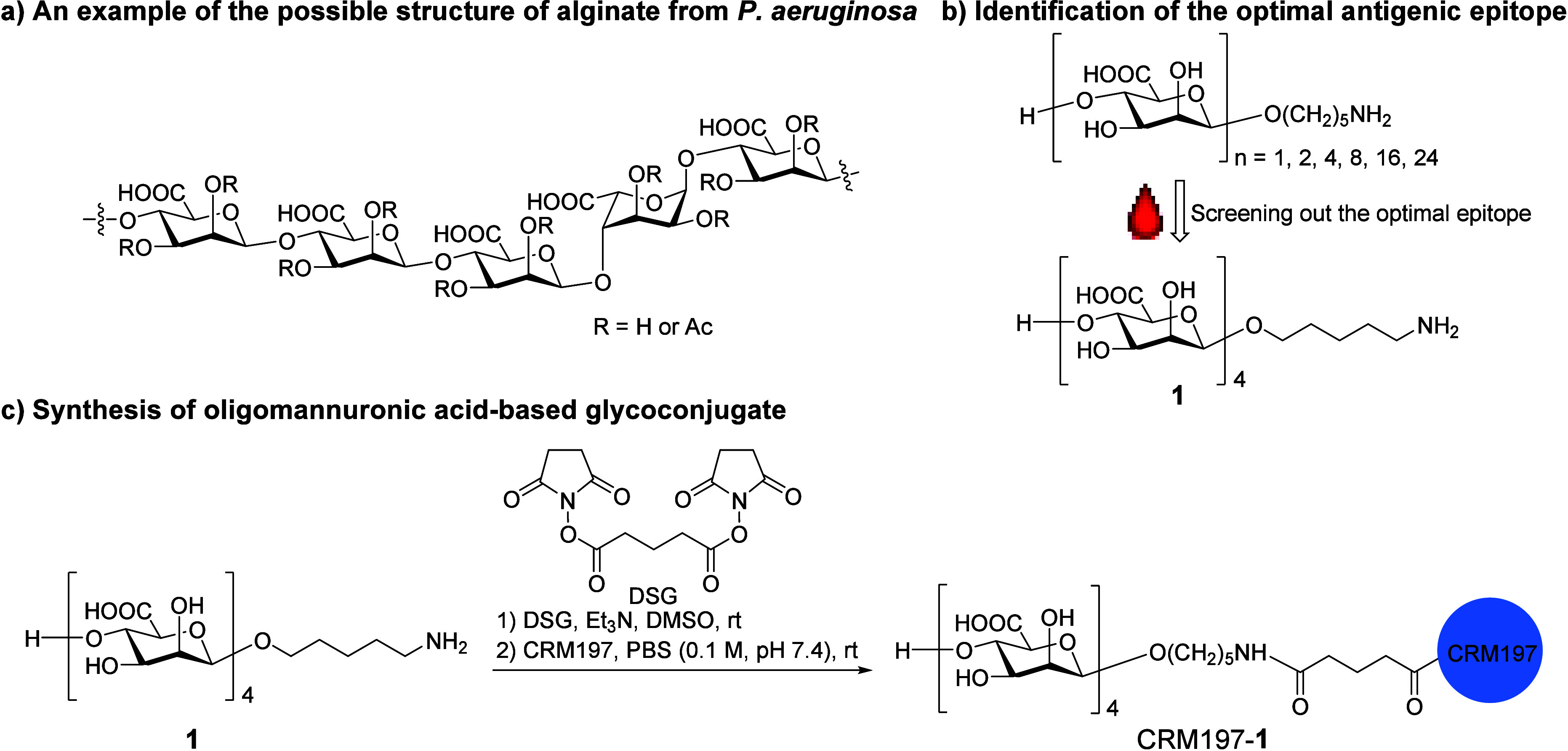
Synthesis of oligomannuronic acid-based CRM197–**1** conjugate based on the identification of the optimal antigenic epitope from a range of synthetic mannuronic acid alginate glycans.

One of the major issues in the development of a successful vaccine against *P. aeruginosa* is the antigenic variability presumably due to the genotypic variation and the multiple serotypes, which may drive the evasion of the host protective immunity.^17^ Thus, development of a broadly protective vaccine for prevention of *P. aeruginosa* infection remains elusive. In contrast to *O*-antigens, alginate is a highly conserved antigen of different strains of *P. aeruginosa*, which holds the potential to provide complete protection against the pathogen.^12,18^ Not only do mucoid strains of *P. aeruginosa* overproduce alginate, but also nonmucoid strains express a small amount of alginate.^19^ As a pivotal component of biofilms, alginate composed of 1,4-linked partially *O*-acetylated β-d-mannuronic acid and α-l-guluronic acid residues is associated with the pathogenesis of chronic pulmonary infection in CF patients (Figure 1a).^20^ Notably, human monoclonal antibodies to alginate that protected mice against both mucoid and nonmucoid strains of *P. aeruginosa* bound well to polymannuronic acid.^21^ Rabbit antisera to polymannuronic acid–flagellin conjugate showed protective efficacy against both mucoid and nonmucoid strains in a murine model of lung infection.^22^ Thus, polymannuronic acid might be a conserved antigen that could induce broadly protective antibodies against almost all strains of *P. aeruginosa*.

Considering the microheterogeneity of polymannuronic acid, the potential contamination of polymannuronic acid by endotoxin during isolation of the antigen from cultured bacteria, the ill-defined conjugation process, and the batch-to-batch variation, preparation of well-defined glycoconjugates against *P. aeruginosa* is challenging. Herein we report a semisynthetic oligomannuronic acid-based glycoconjugate vaccine that is broadly protective against both mucoid and nonmucoid strains of *P. aeruginosa*. The well-defined glycoconjugate vaccine formulated with Freund’s adjuvant (FA) induced a strong and specific immune response, stimulated the production of high titers of opsonic antibodies, and protected mice against infections of both mucoid and nonmucoid types of *P. aeruginosa* strains.

## Results and Discussion

### Preparation and Characterization of Oligomannuronic Acid-Based CRM197–**1** Conjugate

In view of the high ratio of mannuronic acid to guluronic acid in alginate of most *P. aeruginosa* as well as the broad protection of rabbit sera to the nonacetylated polymannuronic acid-based glycoconjugate against both mucoid and nonmucoid strains,^22,23^ we envisioned that the synthetic homogeneous nonacetylated mannuronic acid alginate glycans might serve as the protective epitopes for inducing a strong immune response in vaccinated animals and providing broad protection against both types of *P. aeruginosa*. To this end, the mannuronic acid tetrasaccharide **1** obtained by the bimodal glycosylation of glycosyl ynenoates has been identified as the optimal antigenic epitope for recognition with the sera antibodies from mice immunized with inactivated *P. aeruginosa* from six synthetic mannuronic acid alginate glycans ranging from the monomer to the 24-mer (Figure 1b).^24,25^ We aimed to use the simple and highly conserved synthetic mannuronic acid tetrasaccharide to develop a broadly protective semisynthetic glycoconjugate vaccine candidate against antibiotic-resistant *P. aeruginosa*, overcoming the heterogeneity and potential contamination associated with the isolated glycan antigens.

To recruit T-cell help and increase the immunogenicity of the oligomannuronic acid, the conjugation strategy that proved to be safe and effective against pathogenic bacteria such as *Haemophilus influenzae* type b, *Neisseria meningitidis*, *Streptococcus pneumoniae*, and *Salmonella typhi* was utilized.^26^ By employing the bifunctional glutaryl group, which would not affect the immunological activities of the resulting glycoconjugates as the linker,^27^ the mannuronic acid tetrasaccharide **1** with a pentyl amine at the reducing end, which was synthesized and characterized in our previous work,^24^ was first reacted with di(*N*-succinimidyl)glutarate (DSG) to give the desired activated monoester, which was then subjected to covalent coupling with nontoxic diphtheria toxin mutant CRM197,^28^ a widely used carrier protein in licensed vaccines, in phosphate-buffered saline (PBS, 0.1 M, pH 7.4), to produce the CRM197–**1** conjugate (Figure 1c). As shown in the MALDI-TOF mass spectrometry of the CRM197–**1** and CRM197, it was calculated that an average of 9.7 molecules of tetrasaccharide **1** were covalently coupled to each CRM197 (Figure S1). By using CRM197 as the control, SDS-PAGE analysis of the CRM197–**1** also verified the glycan loading of the tetrasaccharide **1** on CRM197 (Figure S1). In addition, the human serum albumin (HSA)–**1** conjugate was prepared as a capture reagent for detecting the tetrasaccharide **1**-specific antibodies.^24^

### The CRM197–**1** Conjugate Elicited a Strong and Specific Immune Response

**Figure 2:**
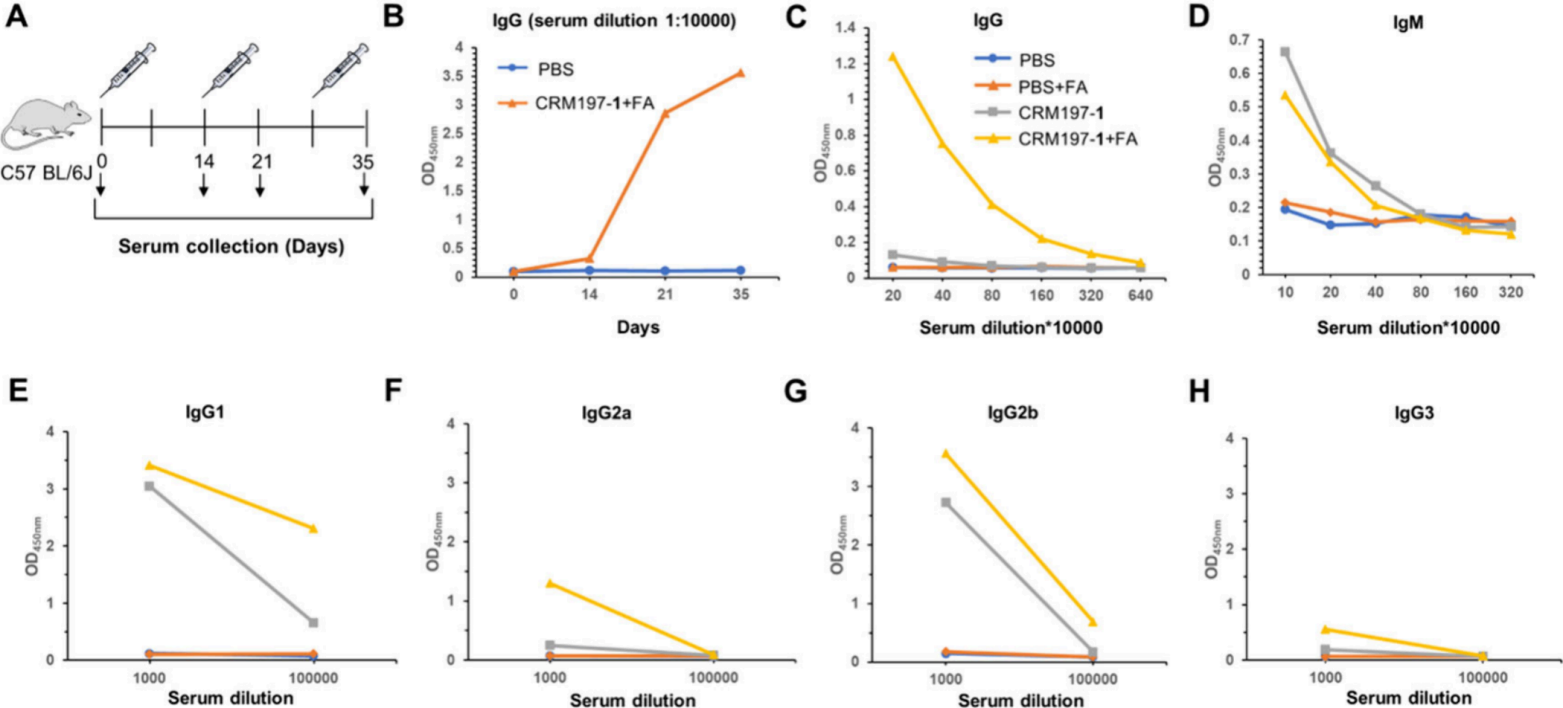
Analysis of antibody titers in sera by ELISA. (A) Immunization schedule. C57BL/6J mice (*n* = 4–6) were immunized subcutaneously on day 0 with CRM197–**1** (0.5 μg sugar per dose) and boosted on days 14 and 28 with CRM197–**1** (2 μg sugar per dose) with or without Freund’s adjuvant (FA). Control mice received only PBS or PBS with FA. (B) IgG titer at different time points (1:10 000 dilution). (C–H) IgG, IgM, and IgG isotypes of day 35 sera analyzed by ELISA. The HSA–**1** conjugate was used as the coating antigen for ELISA. The antibody titers were measured in triplicate and plotted as mean ± SD.

To evaluate the immunogenicity of the CRM197–**1** conjugate, immunization of female C57BL/6J mice was performed subcutaneously with either the CRM197–**1** conjugate mixed with FA or the CRM197–**1** conjugate solely (Figure 2). The mice in the control groups received only PBS or FA in PBS. The immunized groups were vaccinated with one priming dose on day 0, one booster dose on day 14, and another one booster dose on day 28 (Figure 2A). The antibody titers of postimmune sera were determined by ELISA, in which the HSA–**1** conjugate was employed as the coating antigen to detect the tetrasaccharide **1**-specific immune response. As shown in Figure 2B,C, mice immunized with the CRM197–**1** conjugate formulated with FA elicited high titers of tetrasaccharide **1**-specific IgG antibodies on day 35, whereas mice vaccinated with the CRM197–**1** conjugate alone generated low titers of tetrasaccharide **1**-specific IgG antibodies on day 35, indicating that the adjuvant FA played a critical role in the induction of class switch to the T-cell-dependent antigen-specific IgG antibodies.^29^ The control groups of mice that were sham-immunized with FA in PBS or only PBS did not exhibit significant humoral immune response. In the meantime, comparable titers of tetrasaccharide **1**-specific IgM antibodies of day 35 sera were observed with the group treated with the CRM197–**1** conjugate plus FA and those with the CRM197–**1** conjugate alone (Figure 2D).

The immune response in vivo was further assessed by analysis of the IgG isotypes. As depicted in Figure 2E–H, IgG1 and IgG2b antibodies accounted for the majority of the tetrasaccharide **1**-specific IgG antibodies of day 35 sera produced from mice immunized with the CRM197–**1** conjugate plus FA, while a weak IgG2a and IgG3 response was observed. In comparison with mice inoculated with CRM197–**1** and FA, mice vaccinated with the CRM197–**1** conjugate alone generated lower titers of IgG1 and IgG2b antibodies. The sham-immunized groups were unable to elicit significant levels of tetrasaccharide **1**-specific IgG isotypes. These results indicated the class switch to IgG antibodies, especially the high titers of IgG1 and IgG2b antibodies, demonstrating that the CRM197–**1** conjugate formulated with FA induced a strong immune response in mice and produced high titers of T-cell-dependent tetrasaccharide **1**-specific antibodies.^30^

### The Anti-CRM197–**1** Antibodies Recognized *P. aeruginosa* and Mediated the Opsonic Killing of the Bacteria

**Figure 3:**
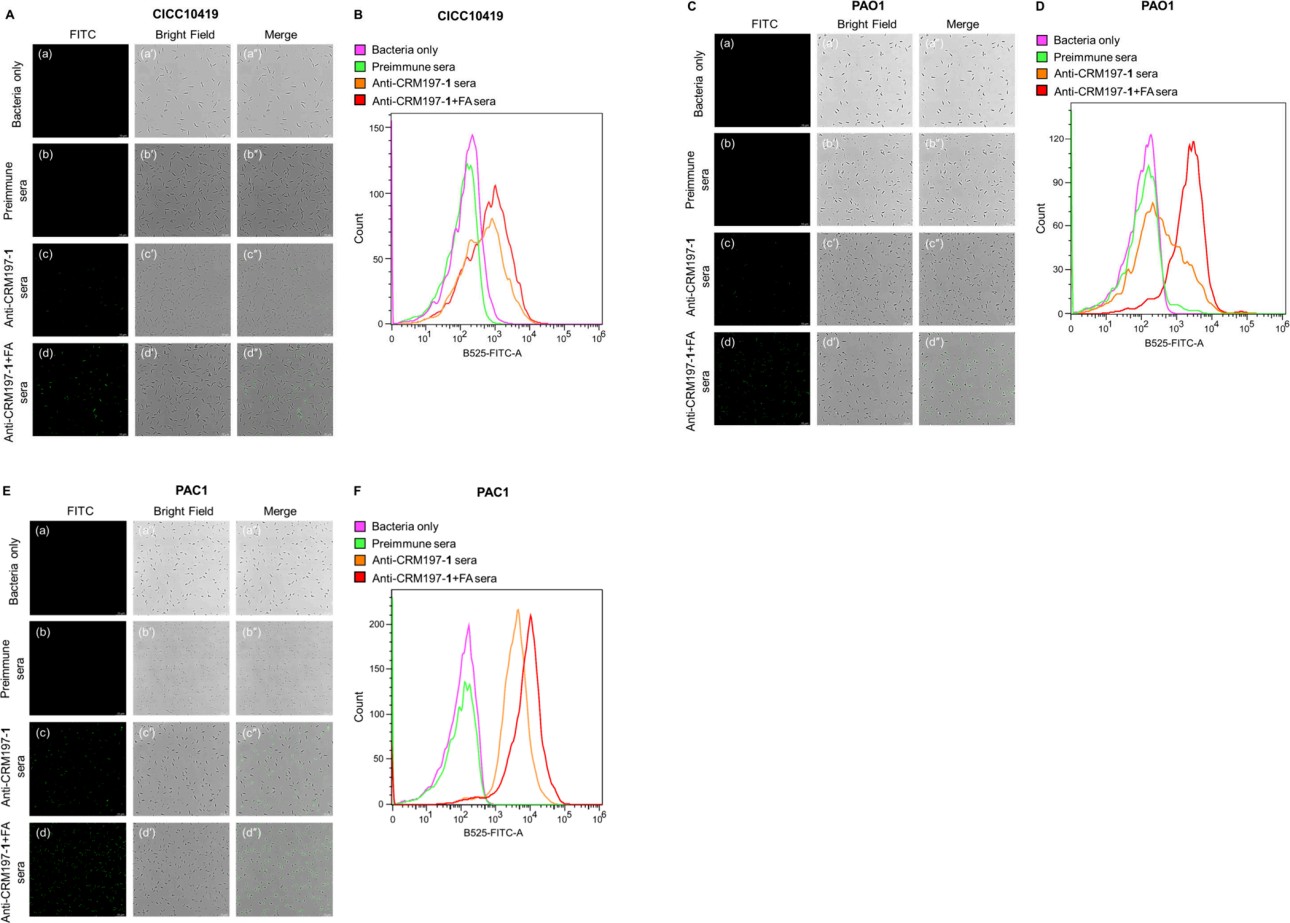
Surface staining of *P. aeruginosa*. (A, C, E) Immunofluorescent staining of the ultraviolet-inactivated *P. aeruginosa* CICC10419 (A), PAO1 (C), and PAC1 (E) using only bacteria without sera treatment (a), using pooled sera from mice without vaccine treatment (b), using pooled sera from mice immunized with CRM197–**1** conjugate without FA (c), and using pooled sera from mice immunized with CRM197–**1** conjugate formulated with FA (d). The associated panels a′–c′ display the bright field images, and the associated panels a″–c′′ display the overlaid images. (B, D, F) The ultraviolet-inactivated *P. aeruginosa* CICC10419 (B), PAO1 (D), and PAC1 (F) were incubated with anti-CRM197–**1**+FA sera, anti-CRM197–**1** sera, or preimmune sera, and surface staining with FITC-labeled goat anti-mouse IgG was analyzed by flow cytometry. Only bacteria cells served as background control.

The binding of the postimmune sera on day 35 from mice immunized with the CRM197–**1** conjugate both with and without FA to the intact *P. aeruginosa* strain was then analyzed by immunofluorescence (IF) assay and flow cytometry (Figure 3). Considering the diversity in alginate production and acetylation of *P. aeruginosa*, different types of *P. aeruginosa* strains including a nonmucoid *P. aeruginosa* CICC 10419 strain with low production of alginate, a typical nonmucoid PAO1 strain with very low production of alginate, and a clinical mucoid PAC1 strain with overproduction of alginate that is usually partially acetylated were selected to test the vaccine’s efficacy (the levels of alginate produced by the bacteria were determined by the alginate assay; Figure S2).^31^ The recognition of the ultraviolet-inactivated nonmucoid *P. aeruginosa* CICC 10419, nonmucoid PAO1, and a clinical mucoid PAC1 by anti-CRM197–**1** antibodies was visualized by confocal laser scanning microscopy (CLSM) in the IF assay (Figure 3A,C,E). The green fluorescein isothiocyanate (FITC)-labeled goat anti-mouse IgG (green) was used as a secondary antibody for immunofluorescence staining. In comparison with the control groups that used the only bacteria without sera treatment and the preimmune sera from mice, the postimmune sera on day 35 from mice immunized with CRM197–**1** conjugate formulated with FA bound significantly to the inactivated *P. aeruginosa* CICC 10419 and PAO1 (see the enlarged images of the (d) and (d″) regions in Figures S3 and S4 of Supporting Information). Remarkably, a very strong binding of the postimmune sera on day 35 from mice immunized with CRM197–**1** conjugate plus FA to the inactivated *P. aeruginosa* PAC1 was observed (see the enlarged images of the (d) and (d″) regions in Figure S5 of Supporting Information), which was probably due to the overproduction of alginate on the surface of the mucoid strain of *P. aeruginosa* PAC1. With regard to the postimmune sera on day 35 from mice immunized with CRM197–**1** conjugate without FA, they showed weak binding to *P. aeruginosa* CICC 10419 and PAO1, but significant binding to PAC1, indicating that FA contributed greatly to the stronger recognition ability of the resulting antibodies against *P. aeruginosa* (see the enlarged images of the (c) and (c″) regions in Figures S3–S5 of Supporting Information). The flow cytometry data that indicated the binding capacity of postimmune sera with *P. aeruginosa* were basically consistent with the results of the IF assays (Figure 3B,D,F). The anti-CRM197–**1**+FA sera on day 35 bound obviously to the inactivated *P. aeruginosa* CICC 10419 and PAO1 compared with the preimmune sera and the background control. Especially, the inactivated *P. aeruginosa* PAC1 was very well recognized by the anti-CRM197–**1**+FA sera on day 35. Although the anti-CRM197–**1** sera on day 35 alone exhibited weak binding to *P. aeruginosa* CICC 10419 and PAO1, they bound strongly to the surface of *P. aeruginosa* PAC1. These results implied that the anti-CRM197–**1** antibodies could recognize the bacterial surface exopolysaccharide of *P. aeruginosa* depending on the amount of alginate expressed by the bacteria as well as the formulation with FA.

**Figure 4:**
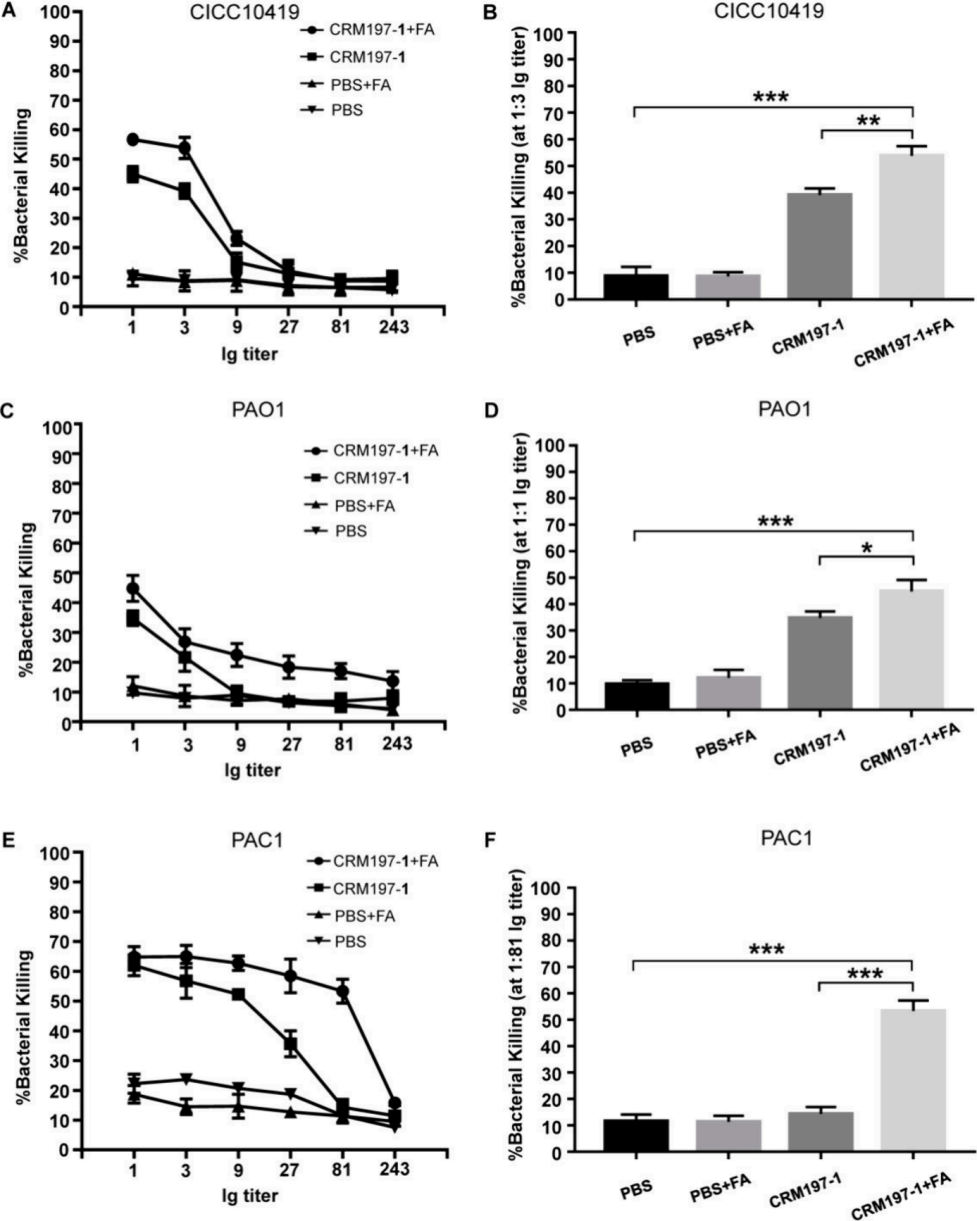
In vitro opsonophagocytic killing activity of postimmune sera against *P. aeruginosa*. (A, C, E) The opsonophagocytic killing assay of *P. aeruginosa* CICC10419 (A), PAO1 (C), and PAC1 (E). (B, D, F) The antibody titer calculated for about 50% or more killing of *P. aeruginosa* CICC10419 (B), PAO1 (D), and PAC1 (F). Data were analyzed by the unpaired *t*-test, and *p* values of <0.05 were considered statistically significant: *, *p* < 0.05; **, *p* < 0.01; and ***, *p* < 0.001.

To determine the functional activity of antibodies raised against CRM197–**1** conjugate, an in vitro opsonophagocytic killing assay (OPKA) was performed (Figure 4). Differentiated HL-60 cells were incubated with *P. aeruginosa* CICC 10419, PAO1, and PAC1 that were preopsonized with anti-CRM197–**1** antibodies or control sera at different dilutions using baby rabbit serum as a complement source. The anti-CRM197–**1**+FA antibodies promoted the complement-mediated phagocytosis against *P. aeruginosa* CICC 10419 and nonmucoid PAO1, although they exhibited modest opsonic killing activity with about a 50% bacterial killing rate at an antibody titer value of 3 and 1, respectively (Figure 4A–D). In contrast, the anti-CRM197–**1**+FA antibodies elicited stronger phagocytic activity against mucoid PAC1 with an above 50% bacterial killing rate at an antibody titer value of 81 (Figure 4E,F). Notably, the anti-CRM197–**1** antibodies alone showed relatively weaker opsonic killing activities against the different strains of *P. aeruginosa* compared with those with the anti-CRM197–**1**+FA antibodies. The CRM197–**1** conjugate administered without FA, despite inducing relatively much lower levels of IgG antibodies than those elicited by CRM197–**1** conjugate plus FA (Figure 2C), still resulted in the production of a considerable amount of functional antibodies through the T-cell-dependent immune response, which could activate the complement and promote the opsonic killing of *P. aeruginosa*. Thus, the CRM197–**1** conjugate alone could induce a protective immune response, even at lower IgG antibody titers. No opsonic killing activity was observed with the control sera. Overall, the opsonic killing activity varied among the different strains of *P. aeruginosa*, and it was significantly stronger for *P. aeruginosa* PAC1 probably due to the overexpression of alginate on the surface of this mucoid strain. The pattern of the opsonic killing activity was consistent with those observed with the alginate-based vaccines against *P. aeruginosa*.^21,22,32^ Furthermore, FA was found to enhance the opsonic killing activities of the resulting antibodies against the different strains of *P. aeruginosa*, suggesting the vital role of FA in strengthening the immunogenicity of the CRM197–**1** conjugate for induction of a stronger protective immune response.

### Vaccination with the CRM197–**1** Conjugate Promoted the Pulmonary and Blood Clearance of *P. aeruginosa* and Improved the Survival Rates of Mice against *P. aeruginosa* Infection

**Figure 5:**
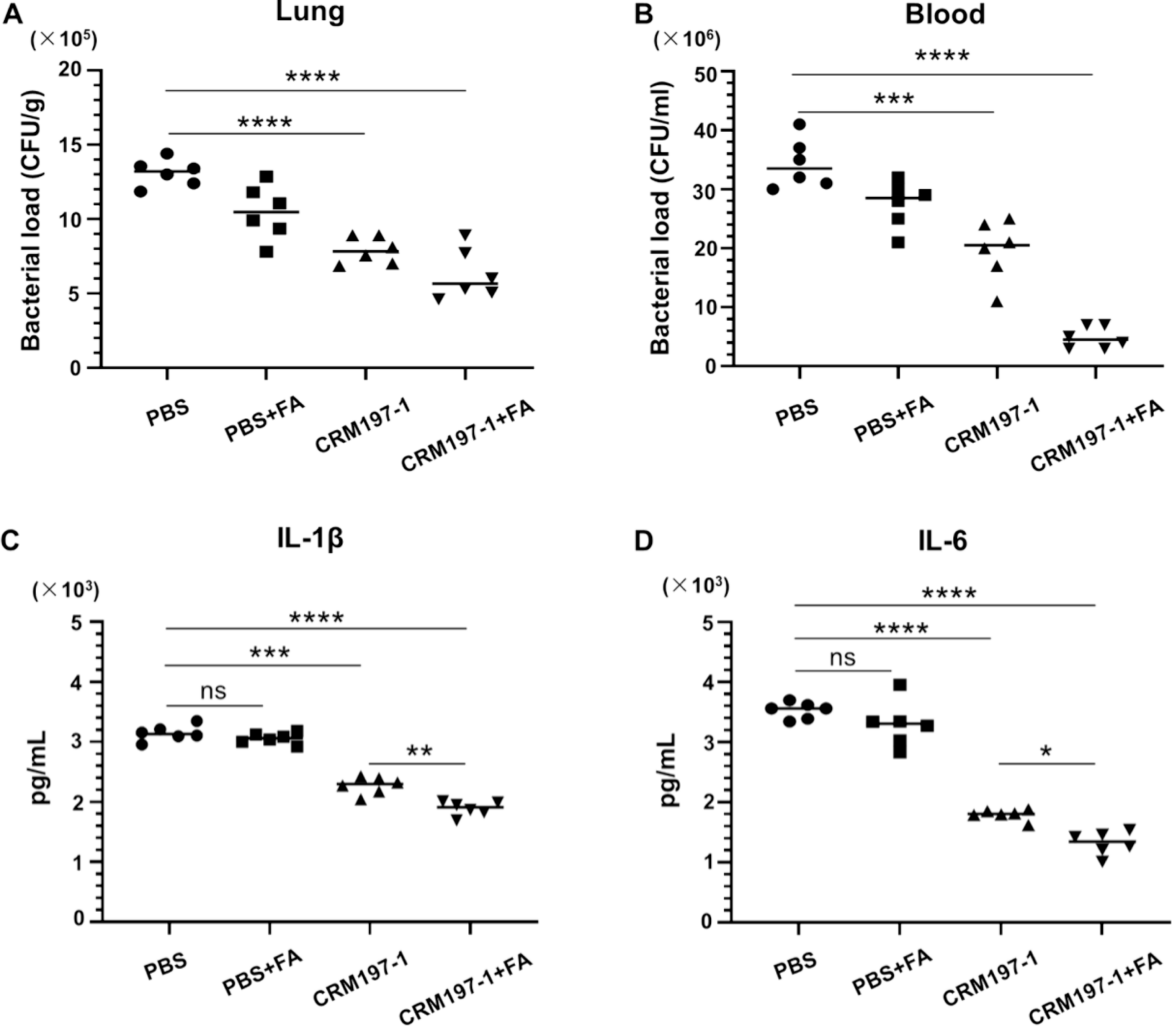
Vaccination with CRM197–**1** conjugate formulated with FA induces protective immunity in mice against the mucoid stain of *P. aeruginosa*. (A, B) Bacterial loads in lungs (A) and blood (B) from vaccinated and control mice 48 h postinfection (*n* = 6 mice/group). Mice were immunized with the CRM197–**1** with or without Freund’s adjuvant, respectively, on day 0, day 14, and day 28 and then challenged by 5 × 10^6^ CFU of strain PAC1 1 week after the final immunization. The control group received PBS with or without Freund’s adjuvant. The data were analyzed by one-way ANOVA with multiple comparisons. *, *p* < 0.05; **, *p* < 0.01; ***, *p* < 0.001; ****, *p* < 0.0001 were considered to be significant as indicated. (C, D) Serum levels of proinflammatory cytokines of IL-1β (C) and IL-6 (D) in vaccinated and control mice 48 h postinfection (*n* = 6 mice/group). Statistical analysis was performed by a two-tailed Student’s *t*-test using Prism software.

As the infection of most mucoid strains of *P. aeruginosa* is not lethal in mice unless very high doses are applied,^22^ a clearance model was used to evaluate the protective efficacy of the CRM197–**1** conjugate vaccine against mucoid *P. aeruginosa* PAC1 strain in vivo (Figure 5). Mice were immunized on day 0, 14, and 28 according to the previous immunization protocol and infected with 5 × 10^6^ colony forming units (CFU) of *P. aeruginosa* PAC1 strain via intratracheal instillation 1 week after final immunization. The effect of vaccination on bacterial load was determined by quantifying viable bacteria in the lung and blood of both immunized and control groups. Vaccination with CRM197–**1** and FA significantly enhanced the clearance of the mucoid PAC1 strain of *P. aeruginosa* from the lung and blood of mice after 48 h of infection compared with the control groups receiving PBS or FA (Figure 5A,B). Thus, active immunization of CRM197–**1** elicited a protective immune response that resulted in a reduction of bacteria load in lung and blood against mucoid strains. To determine whether immunization with CRM197–**1** could mitigate cytokine release during the infection of *P. aeruginosa* PAC1 strain, proinflammatory cytokines IL-1β and IL-6 were measured at serum levels.^33^ Compared with the control group, the immunized mice showed markedly lower levels of IL-1β and IL-6 in their sera (Figure 5C,D). These findings suggested that the CRM197–**1** conjugate vaccine may prevent the release of proinflammatory cytokines and effectively attenuate the inflammation caused by *P. aeruginosa* infection in mice.

**Figure 6:**
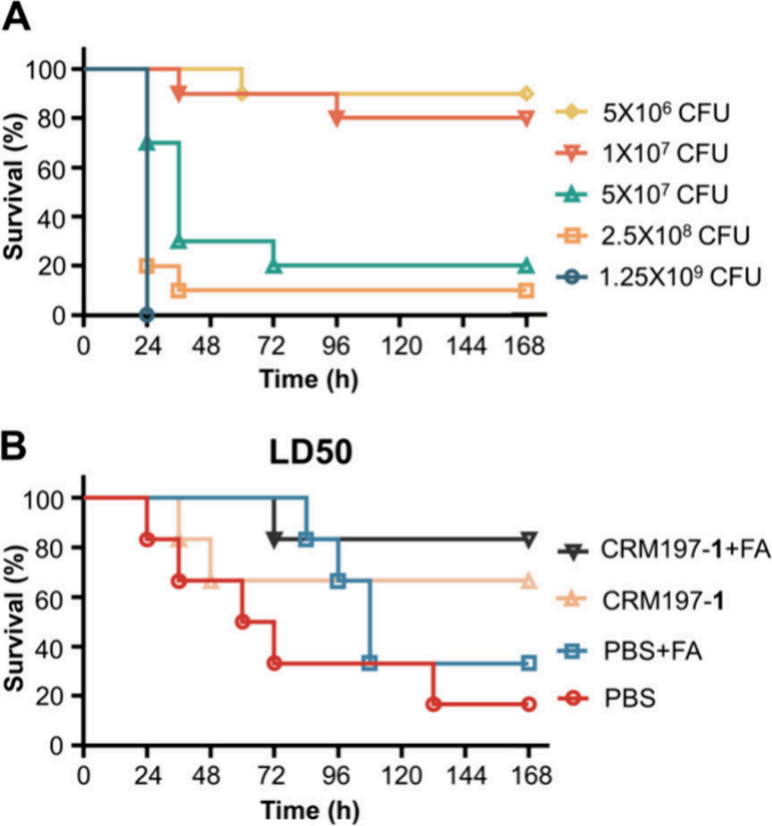
Survival of mice immunized with CRM197–**1** conjugate formulated with FA following challenge with the nonmucoid PAO1 strain of *P. aeruginosa*. (A) Mice (*n* = 10 mice/group) were infected with the indicated dose of PAO1 by intratracheal instillation administration, and survival of the mice was recorded for 7 days. (B) Mice (*n* = 6 mice/group) were immunized with the CRM197–**1** with or without Freund’s adjuvant, respectively, on day 0, day 14, and day 28 and then challenged with PAO1 strain (2.3 × 10^7^ CFU/mouse) 1 week after the final immunization. The control group received PBS with or without Freund’s adjuvant. Statistical significance of differences between groups was analyzed by the log rank test. Log-rank test: PBS versus CRM197–**1**/FA, *p* = 0.02. Difference associated with a *p* value of less than 0.05 was considered as statistically significant.

Evaluation of the protection efficacy of the CRM197–**1** conjugate vaccine against the nonmucoid strain of *P. aeruginosa* in vivo was carried out through the challenge experiment with nonmucoid PAO1 strain using an acute lethal pneumonia model (Figure 6).^34^ Before the challenge study, we determined the LD50 (50% of the lethal dose) value of the PAO1 strain in C57/BL6 mice by intratracheal instillation of different doses of the bacteria. Based on the mortality rate after infection of mice with 5 × 10^6^, 1 × 10^7^, 5 × 10^7^, 2.5 × 10^8^, and 1.25 × 10^9^ CFU of the PAO1 strain (*n* = 10 mice/group), the LD50 was calculated to be 2.3 × 10^7^ CFU using SPSS 27.0 software (Figure 6A).^35^ Similar to the previous immunization protocol, mice were immunized on day 0, 14, and 28, challenged with PAO1 strain via intratracheal instillation 1 week after final immunization, and monitored for another 7 days (*n* = 6 mice/group). Vaccination with CRM197–**1** and FA provided 83.3% protection for mice infected with 2.3 × 10^7^ CFU (LD50) of the PAO1 strain, whereas only 16.7% of mice immunized with PBS survived the same challenge (Figure 6B). In addition, the survival rates for the CRM197–**1** group and the PBS/FA group were 66.7% and 33.3%, respectively. Overall, these results revealed that vaccination with CRM197–**1**/FA significantly protected the mice against nonmucoid PAO1 infection compared with those of the PBS group (*p* < 0.05). The effective protection of mice against the nonmucoid PAO1 strain might be due to the rapid production of alginate in vivo by the nonmucoid isolate.^21^

## Conclusions

In summary, we have developed a broadly protective semisynthetic glycoconjugate vaccine against both mucoid and nonmucoid strains of *P. aeruginosa*. By utilizing the readily available synthetic mannuronic acid tetrasaccharide **1** as the single conserved antigen instead of heterogeneous high-molecular-weight alginate or polymannuronic acid, a well-defined CRM197–**1** conjugate was prepared and characterized as an antibacterial vaccine, which elicited a specific T-cell-dependent immune response and high titers of opsonic antibodies with the assistance of FA. The resulting antibodies not only bound to *P. aeruginosa* with strong recognition to the alginate-overproducing mucoid strain but also mediated the opsonic killing of *P. aeruginosa*, especially for the clinical mucoid isolate PAC1. Notably, FA played a significant role in generating high titers of antigen-specific IgG antibodies, improving antibody recognition ability, and enhancing opsonic killing activities against the different strains of *P. aeruginosa*. Vaccination of CRM197–**1** formulated with FA protected mice against both mucoid and nonmucoid strains of *P. aeruginosa* by reducing bacteria load in the lung and blood, lowering the release of proinflammatory cytokines, and improving the survival rates. The semisynthetic CRM197–**1** conjugate may serve as a promising cost-effective vaccine that provides broad protection against infections of virtually all strains of *P. aeruginosa*.

## Supporting Information Available

Materials and methods including preparation of the glycoconjugates, immunization experiment, ELISA assay, alginate assay, immunofluorescence assay, flow cytometry, in vitro opsonophagocytic killing assay, clearance experiment, and challenge experiment; Figures S1–S5 (PDF)
