# Supplementary material for: A Semisynthetic Oligomannuronic Acid-Based Glycoconjugate Vaccine against Pseudomonas aeruginosa
Source: ACS Cent Sci. 2024 Jul 10;10(8):1515–23. doi: 10.1021/acscentsci.4c00387 (PMC11363335; doi:10.1021/acscentsci.4c00387)
Supplement: Supplementary file 1 — oc4c00387_si_001.pdf [file oc4c00387_si_001.pdf]

Supporting Information

A Semisynthetic Oligomannuronic Acid-Based Glycoconjugate Vaccine against *Pseudomonas aeruginosa*

Yiyue Zhang,[1†] Xiaotong Wang,[1†] Youling Liang,[1] Liangliang Zhang,[1] Jiahao Fan,[2] and You Yang[1,2]*

[1]Shanghai Frontiers Science Center of Optogenetic Techniques for Cell Metabolism, Shanghai Key Laboratory of New Drug Design, School of Pharmacy, East China University of Science and Technology, 130 Meilong Road, Shanghai 200237, China.

*E-mail: yangyou@ecust.edu.cn

[2]Engineering Research Center of Pharmaceutical Process Chemistry, Ministry of Education, East China University of Science and Technology, 130 Meilong Road, Shanghai 200237, China.

[†]Y.Z. and X.W. contributed equally to this work.

Contents

	Page
1. Materials and methods	S3
2. References	S11
3. Figure S1	S12
4. Figure S2	S13
5. Figure S3	S14
6. Figure S4	S15
7. Figure S5	S16

1. Materials and methods

1.1 Bacterial strains and mice

The *Pseudomonas aeruginosa* strain CICC10419 was purchased from CICC (China Center of Industrial Culture Collection). The *Pseudomonas aeruginosa* PAO1 (BNCC360090) was purchased from BNCC (BeNa Culture Collection, China). The *Pseudomonas aeruginosa* PAC1 (a clinical isolate) was provided by Huashan Hospital, Fudan University, China. Bacteria were preserved at –80 °C in nutrient-rich broth (NB) containing 25% glycerol. The glycerol-frozen strains were revived on NB agar plate, and a selected single colony was incubated in NB solution at 37 °C for 12 h at 200 rpm on a shaker. Six to eight-week-old female C57BL/6J mice were purchased from Jiesijie, Shanghai, China. The in vivo experiments were conducted with mice under the ethics certificate ECUST-2022-032 of East China University of Science and Technology. All mice were kept under specific pathogen-free conditions during experiments. All efforts were made to minimize animal suffering.

1.2 Conjugation of tetrasaccharide 1 to CRM197

To a solution of di(*N*-succinimidyl) glutarate (DSG; 8.4 mg, 25.5 μmol; 97% purity; J＆K Scientific, China) and triethylamine (8 μL, 0.06 mmol; Shanghai Aladdin Biochemical Technology Co., Ltd) in anhydrous dimethyl sulfoxide (DMSO; 160 μL; 99.9% purity; Beyotime Biotechnology, China) was added dropwise a solution of tetrasaccharide **1** (2.06 mg, 2.55 μmol), which was synthesized and characterized in the previous reference,[1] in anhydrous DMSO (80 μL) at room temperature. After being stirred at room temperature for 2 h, the solution was treated with phosphate buffer saline (PBS; 100 mM, pH = 7.4, 320 μL; Biosharp, China). The mixture was then extracted with chloroform ($CHCl_3$; 5 mL; 99% purity, Sinopharm Chemical Reagent Co., Ltd, China), separated by centrifugation (1800 g, 2 min; Heraeus Multifuge X1R, Thermo Fisher Scientific), and the organic phase was discarded. The extraction procedure was repeated three times. The organic phase was further removed from the aqueous layer by high-speed centrifugation (14500 g, 1 min; Sorvall Legend Micro 17R, Thermo Fisher Scientific) and this step was repeated three

times. The isolated aqueous phase solution was added to a solution of CRM197 (Native Diphtheria Toxin CRM197, Mutant [Glu52]; 1 mg, 17.3 nmol; Creative BioMart, USA) in PBS (100 mM, pH = 7.4, 1 mL). The mixture was stirred at room temperature for 18 h and dialyzed against distilled water three times using a centrifugal filter (30 kDa MWCO, Millipore, USA). This procedure was repeated three times and finally concentrated to 200 μL. The average molecular weight of the glycoconjugate was characterized by MALDI-TOF mass spectrometry (4800 plus, AB SCIEX, USA) using CRM197 as a standard (Figure S1). Based on the MALDI-TOF analysis of the CRM197-**1** conjugate and CRM197, the CRM197-**1** conjugate contained an average of 9.7 molecules of tetrasaccharide **1** on each CRM197. The glycoconjugate was prepared in 5X SDS-PAGE sample loading dye (Beyotime Biotechnology, China) and resolved by 10% SDS-PAGE (Figure S1). The electrophoresis was performed in electrophoresis buffer at 120 V and 25 mA for 90 min and the gel was stained by PageBlue protein staining solution (Thermo Fisher Scientific).

1.3 Conjugation of tetrasaccharide 1 to HSA

To a solution of di(*N*-succinimidyl) glutarate (DSG; 7.3 mg, 22.5 μmol) and triethylamine (7 μL, 0.05 mmol) in anhydrous DMSO (160 μL) was added dropwise a solution of tetrasaccharide **1** (1.82 mg, 2.25 μmol) in anhydrous DMSO (80 μL) at room temperature. After being stirred at room temperature for 2 h, the solution was treated with phosphate buffer saline (PBS; 100 mM, pH = 7.4, 320 μL). The mixture was then extracted with chloroform ($CHCl_3$; 5 mL), separated by centrifugation (1800 g, 2 min), and the organic phase was discarded. The extraction procedure was repeated three times. The organic phase was further removed from the aqueous layer by high-speed centrifugation (14500 g, 1 min) and this step was repeated three times. The isolated aqueous phase solution was added to a solution of Human Serum Albumin (HSA; 1 mg, 15 nmol; 95% purity; Sino Biological Inc, China) in PBS (100 mM, pH = 7.4, 1 mL). The mixture was stirred at room temperature for 18 h and dialyzed against distilled water three times using a centrifugal filter (30 kDa MWCO,

Millipore, USA). The average molecular weight of the glycoconjugate was characterized by MALDI-TOF mass spectrometry using HSA as a standard. Based on the MALDI-TOF analysis of the HSA-**1** conjugate and HSA, the HSA-**1** conjugate contained an average of 3.5 molecules of tetrasaccharide **1** on each HSA.[1] The glycoconjugate was prepared in 5X SDS-PAGE sample loading dye and resolved by 10% SDS-PAGE. The electrophoresis was performed in an electrode buffer at 120 V and 25 mA for 90 min and the gel was stained by PageBlue protein staining solution.[1]

1.4. Immunization of mice with the CRM197-1 conjugate

Immunization of six to eight-week-old female C57BL/6J mice (n = 4-6) was performed subcutaneously (s.c.) with the CRM197-**1** conjugate. Six to eight-week-old female C57BL/6J mice (n = 4-6) were immunized with CRM197-**1** conjugate by subcutaneous injection (s.c.). Each mouse received three doses of vaccine in a final volume of 100 μL for each dose. The mice were immunized with the glycoconjugate containing 0.5 μg of antigen **1** emulsified with or without 1:1 (v/v) complete Freund’s adjuvant (CFA, InvivoGen, France) on day 0, and boosted twice with the glycoconjugate containing 2 μg of antigen **1** emulsified with or without 1:1 (v/v) incomplete Freund’s adjuvant (IFA, InvivoGen, France) on days 14 and 28. The control mice received either only PBS or FA in PBS. On days 0, 14, 21, and 35, blood samples were collected by retro-orbital venous plexus puncture using capillary tubes. Sera were obtained from clotted blood samples by centrifugation at 4 °C for 10 min, and stored at –80 ℃.

1.5 ELISA

Costar high-binding polystyrene 96-well plates (Corning, USA) were coated with the HSA-**1** conjugate at a concentration of 10 μg/ml in sodium carbonate-sodium hydrogen carbonate buffer solution (Na_2CO_3-$NaHCO_3$, 0.05 M, pH = 9.6; Shanghai Yuanye Bio-Technology Co., Ltd) at 4 ℃ for 20 h. The plates were washed three times with PBS containing 0.1% Tween-20 (PBS-T; Tween-20 was purchased from Shanghai Macklin Chemical Technology Co., Ltd) and blocked with 2% BSA-PBS

(BSA was purchased from Shanghai Maokang Biotechnology Co., Ltd) at 37 ℃ for 1 h. After washing with PBS-T three times, primary antiserum dilutions in 1% BSA-PBS were added (100 μL per well) and the plates were incubated at 37 ℃ for 2 h. The plates were washed with PBS-T three times and further incubated with a 1:250 diluted secondary antibody in 1% BSA-PBS at 37 ℃ for 1 h in the dark. The secondary antibodies used here were HRP-conjugated goat anti-mouse IgG (Applygen Technologies Inc, China), IgM (Applygen Technologies Inc, China), IgG1 (Applygen Technologies Inc, China), IgG2a (Applygen Technologies Inc, China), IgG2b (Applygen Technologies Inc, China) and IgG3 (Applygen Technologies Inc, China). The plates were washed with PBS-T five times, developed with TMB substrate (Solarbio, China) at 37 ℃ for 20 min, and stopped with 2% sulfuric acid (Solarbio, China). The absorbance values were recorded at 450 nm wavelength with a microplate reader (Synergy2, BioTek, USA).

1.6 Alginate assay

The alginate assay was performed based on a borate/carbazole method described previously.[2] *P. aeruginosa* (CICC10419, PAO1, and PAC1) were grown at 37 ℃ in NB broth (20 mL) for 24 h with shaking (200 rpm). Bacterial cells were removed from a 5 mL bacterial culture by centrifugation (9000 rpm, 3 min, rt). The alginate in the culture supernatant was precipitated by 2% cetylpyridinium chloride (w/v, 5 mL; 98% purity, Shanghai Aladdin Biochemical Technology Co., Ltd.) followed by centrifugation (5000 rpm, 15 min, rt). The pellet was resuspended in 1 M NaCl (5 mL) and reprecipitated with cold (–20 ℃) 2-propanol (5 mL; 99.5% purity, Sinopharm Chemical Reagent Co., Ltd, China) followed by centrifugation (5000 rpm, 15 min, rt). The final alginate pellet was dissolved in distilled water (5 mL). OD_{600} values of bacterial suspensions were measured for normalization. The levels of alginate were analyzed using a borate/carbazole method with a standard curve constructed with the use of sodium alginate (J＆K Scientific, China) in the range 0–100 μg mL^{-1}.

1.7 Immunofluorescence of the ultraviolet-inactivated *P. aeruginosa*

P. aeruginosa (CICC10419, PAO1, and PAC1) were cultured at 37 ℃ under microaerophilic conditions (3-5% O_2-10% CO_2) and harvested by centrifugation (10000 rpm, 5 min, rt). The bacteria were washed with PBS, harvested by centrifugation, and suspended in PBS to approximately 4×10^8 colony-forming units (CFU)/mL. Inactivation of the bacteria was performed by irradiation (λ = 254 nm) at room temperature for 10 min. Cells were washed with PBS, harvested by centrifugation (10000 rpm, 5 min, rt), and frozen at approximately 8×10^8 CFU/mL in fresh NB mixed with 20% (v/v) glycerol at −20 °C.

The bacteria were thawed, harvested by centrifugation (10000 rpm, 5 min, rt), and washed with PBS-T two times. Cells were treated with 3% BSA-PBS blocking buffer at 37 ℃ for 1 h. After washing with PBS-T three times, the samples were incubated with 1:10 diluted antisera or normal sera in 0.5% BSA-PBS at 4 ℃ overnight. After washing with PBS-T three times, the samples were incubated with FITC-labeled goat anti-mouse IgG (1:100 diluted in 1% BSA-PBS, Elabscience Biotechnology Co., Ltd, China) at 37 ℃ for 1 h. After washing with PBS three times, fluorescently labeled bacteria were visualized by a Laser Scanning Confocal Microscope (LSCM, Leica SP8, Germany). Images were processed using the LAS X Office software (Leica SP8, Germany).

1.8 Surface staining of *P. aeruginosa* by flow cytometry

The ultraviolet-inactivated *P. aeruginosa* CICC1041, PAO1, and PAC1 were thawed, harvested by centrifugation (10000 rpm, 5 min, rt), and washed with PBS-T two times. Cells were treated with 3% BSA-PBS blocking buffer at 37 ℃ for 1 h.-After blocking, the samples were washed by PBS-T three times and incubated with 1:10 diluted antisera or normal sera in 0.5% BSA-PBS at 4 ℃ overnight. On the next day, cells were washed with PBS-T three times and incubated with FITC-labeled goat anti-mouse IgG antibody (1:100 diluted in 1% BSA-PBS, Elabscience Biotechnology Co., Ltd, China) at 37 ℃ for 1 h. Again, cells were washed with PBS three times and analyzed by flow cytometry (BD FACSAria, USA).

1.9 In vitro opsonophagocytic killing assay

The opsonophagocytic killing assays (OPKA) were performed as described before.[3] HL-60 cells (Cell Bank/Stem Cell Bank, Chinese Academy of Sciences) were cultured in RPMI1640 medium (Wuhan Pricella Biotechnology Co., Ltd, China) containing 10% fetal bovine serum (FBS; Wuhan Pricella Biotechnology Co., Ltd, China) and 1% penicillin-streptomycin solution (Thermo Fisher Scientific) at 37 ℃ in the presence of 5% CO_2. The HL-60 cells (4×10^5 cells/mL) were seeded and differentiated with 0.8% *N*,*N*-dimethylformamide (DMF; 99.8% purity; Thermo Fisher Scientific) for five days at 37 ℃ in the presence of 5% CO_2 before performing the assay. On day five, cells were harvested by centrifugation (200 g, 5 min). Viable cells were counted using 0.4% trypan blue exclusion (Thermo Fisher Scientific) and resuspended in opsonization buffer B (OBB) at a density of 1×10^7 cells/mL. OBB was a mixture of 40 ml of sterile water, 5 ml of 10X HBSS (with Ca^{2+} and Mg^{2+}; Thermo Fisher Scientific), 5 ml of 1% gelatin (Shanghai Yuanye Bio-Technology Co., Ltd), and 2.7 ml of defined FBS (inactivated for 30 minutes at 56 ℃).

The postimmune sera were diluted in a 3-fold gradient with OBB, setting a total of 8 dilutions (from 1:1 to 1:2187), resulting in a final volume of 20 μL of diluted sera per well of U-bottom 96-well plates (Corning, USA). Then 10 μL of *Pseudomonas aeruginosa* bacterial solution (2×10^5 CFU/mL) was added to the diluted sera, and the solution was shaken (700 rpm, 30 min) for sufficient binding of the bacterial solution to the antibodies in the sera. Baby rabbit serum (10 μL; Pel-Freez, New Zealand) was used as a complement source. Heat-inactivated complement/HL-60 mixture (v/v, 1:4) and active complement/HL-60 mixture (v/v, 1:4) were prepared. A 50 μL of heat-inactivated complement/HL-60 mixture was added to each well in column 1 of U-bottom 96-well plates, and a 50 μL of active complement/HL-60 mixture was added to each well in columns 2-8. Finally, the mixture was incubated for 45 min at 37 ℃ and 5% CO_2 with intermittent shaking for phagocytosis. Each sample was used in triplicate. Preimmune sera (day 0) and PBS-immunized sera were used as negative controls. The viable *P. aeruginosa* were counted on NB agar plates. The percentage killing of *P. aeruginosa* was analyzed relative to the control group lacking antisera.

1.10 Promotion of pulmonary clearance of mucoid strain of *P. aeruginosa*

Six to eight-week-old female C57BL/6J mice were immunized according to the previously described immunization procedure (section 1.4), and *P. aeruginosa* infection was performed one week after the final immunization. *P. ae*ruginosa (clinical PAC1) was cultured in NB medium resuspended in sterile PBS and diluted to 1×10^8 CFU/mL. Mice were fasted for 12 h before anesthesia, intraperitoneally injected with sodium pentobarbital (50 mg/kg; Sigma-Aldrich, USA), and infected through tracheal drip using a microinjection needle (50 μL/dose; 5×10^6 CFU). The mice were monitored every 12 h for changes in weight, feeding activity, mental status, and hair color. The mice were anatomized by intraperitoneal injection of sodium pentobarbital (50 mg/kg) after 48 h of infection. Blood and lungs were collected and homogenized in sterile PBS. Serial dilutions of homogenized contents were spread-plated on NB agar, and the CFU were counted after 12 h post incubation at 37 ℃. Serum levels of interleukin-1β (IL-1β) and IL-6 were determined in the mice at 48 h post-infection of *P. aeruginosa* (clinical PAC1) using mouse ELISA kits (Elabscience Biotechnology Co., Ltd, China).

1.11 Protection against nonmucoid strain of *P. aeruginosa* in an acute murine lung infection model

An acute lethal pneumonia infection model was used to measure the protective efficacy of the CRM197-**1** conjugate vaccine against the nonmucoid PAO1 strain of *P. aeruginosa*. By infection of mice with serially diluted bacterial suspension, the LD50 (50% of the lethal dose) value of PAO1 strain in C57/BL6 mice was calculated using SPSS 27.0 software (IBM, USA). The PAO1 strain was grown overnight in NB with shaking at 37 ℃, centrifuged, washed (PBS), and diluted to 4.6×10^8 CFU/mL. Before infection of mice, PAO1 was plated on NB agar plates to determine the inoculum. Six to eight-week-old female C57BL/6 mice were immunized according to the previously described immunization procedure (section 1.4). One week after the final immunization, mice were fasted for 12 h and anesthetized by intraperitoneal

injection of sodium pentobarbital (50 mg/kg). As for infection, mice were challenged with 50 μL (2.3 × 10^7 CFU; LD50) of PAO1 suspension by intratracheal instillation (22G Introcan Safety IV Catheter). The survival rate of mice was observed twice daily for 7 days. Any of the mice found moribund were euthanized and counted as dead.

1.12 Statistical analysis

Statistical analysis was performed using GraphPad Prism version 9.5 software (GraphPad Software Inc., USA). The antibody titer and opsonophagocytic killing assay were analyzed by unpaired t-test. The CFU data were analyzed by one-way ANOVA with multiple comparisons. The log-rank test was used for survival analysis. *, $p<0.05$; **, $p<0.01$ and ***, $p<0.001$ as indicated.

2. References

1. Zhang, L.; Zhang, Y.; Hua, Q.; Xu, T.; Liu, J.; Zhu, Y.; Yang, Y. Promoter-controlled synthesis and antigenic evaluation of mannuronic acid alginate glycans of *Pseudomonas aeruginosa*. *Org. Lett.* **2022**, *24*, 8381–8386.
2. (a) Mathee, K.; Ciofu, O.; Sternberg, C.; Lindum, P. W.; Campbell, J. I. A.; Jensen, P.; Johnsen, A. H.; Givskov, M.; Ohman, D. E.; Molin, S.; Høiby, N.; Kharazmi, A. Mucoid conversion of *Pseudornonas aeruginosa* by hydrogen peroxide: a mechanism for virulence activation in the cystic fibrosis lung. *Microbiology* **1999**, *145*, 1349–1357. (b) Min, K. B.; Lee, K.-M.; Oh, Y. T.; Yoon, S. S. Nonmucoid conversion of mucoid *Pseudomonas aeruginosa* induced by sulfate-stimulated growth. *FEMS Microbiol. Lett.* **2014**, *360*, 157–166. (c) Knutson, C. A.; Jeanes, A. A new modification of carbazole analysis: application to heteropolysaccharides. *Anal. Biochem.* **1968**, *24*, 470–481.
3. Emmadi, M.; Khan, N.; Lykke, L.; Reppe, K.; Parameswarappa, S. G.; Lisboa, M. P.; Wienhold, S.-M.; Witzenrath, M.; Pereira, C. L.; Seeberger, P. H. A *Streptococcus pneumoniae* type 2 oligosaccharide glycoconjugate elicits opsonic antibodies and is protective in an animal model of invasive pneumococcal disease. *J. Am. Chem. Soc.* **2017**, *139*, 14783−14791.

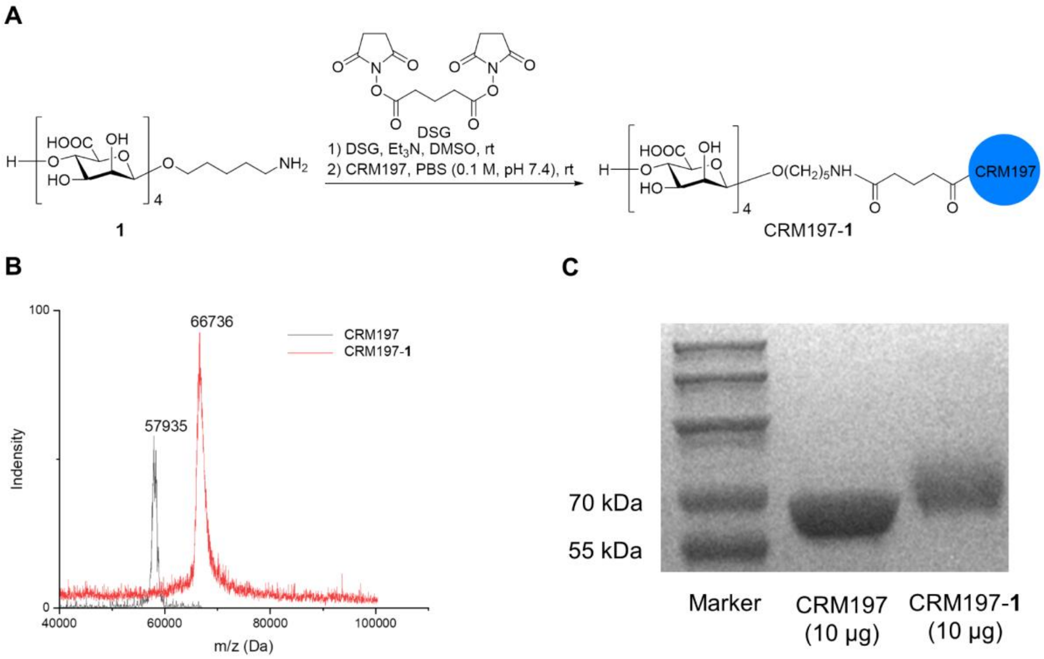

Figure S1. Preparation and characterization of the CRM197-**1** conjugate. (A) Conjugation of tetrasaccharide **1** to carrier protein CRM197 using di(*N*-succinimidyl) glutarate (DSG) as a coupling reagent. (B) MALDI-TOF analysis of the average molecular weight of the CRM197-**1** conjugate using CRM197 as a standard. (C) SDS-PAGE analysis of the CRM197-**1** conjugate and CRM197 using a PageBlue protein staining solution for staining. The molecular weight of the marker bands is indicated at the left.

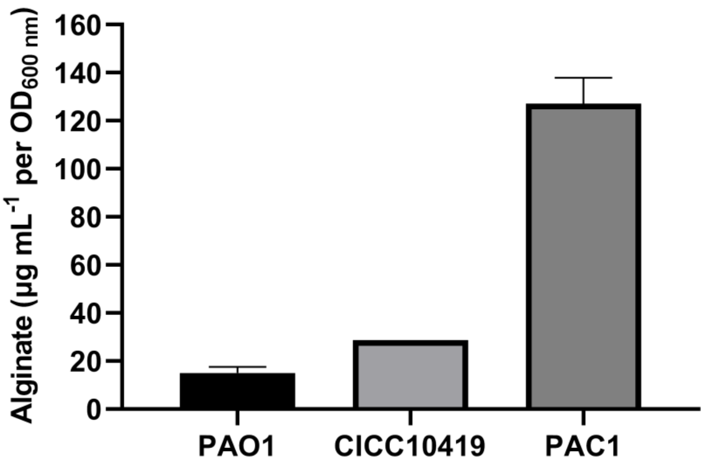

Figure S2. Levels of alginate produced by *P. aeruginosa* PAO1, CICC10419, and PAC1 strains. The amount of alginate was measured in triplicate and plotted as mean ± SD.

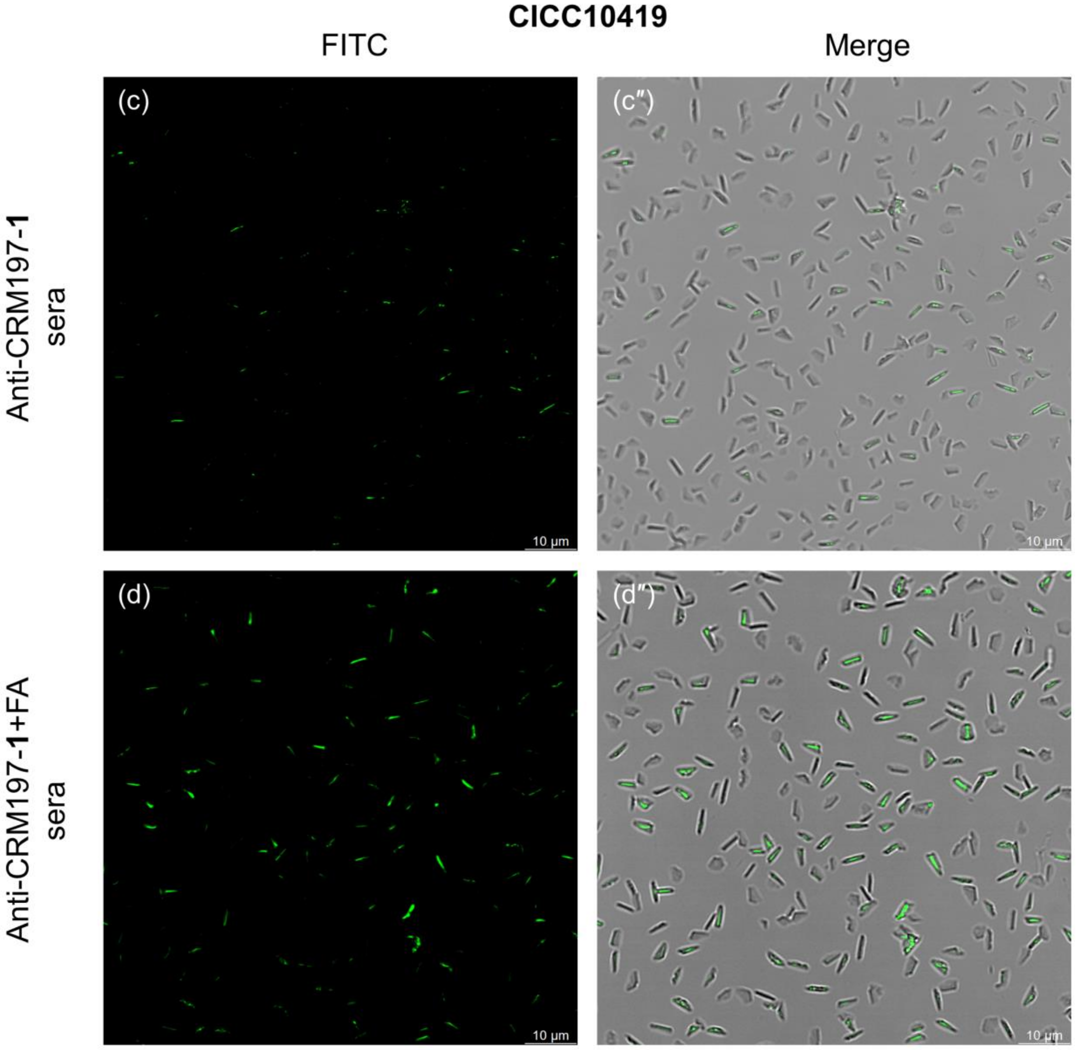

Figure S3. The enlarged images of the (c), (c"), (d), and (d") regions of Figure 3A.

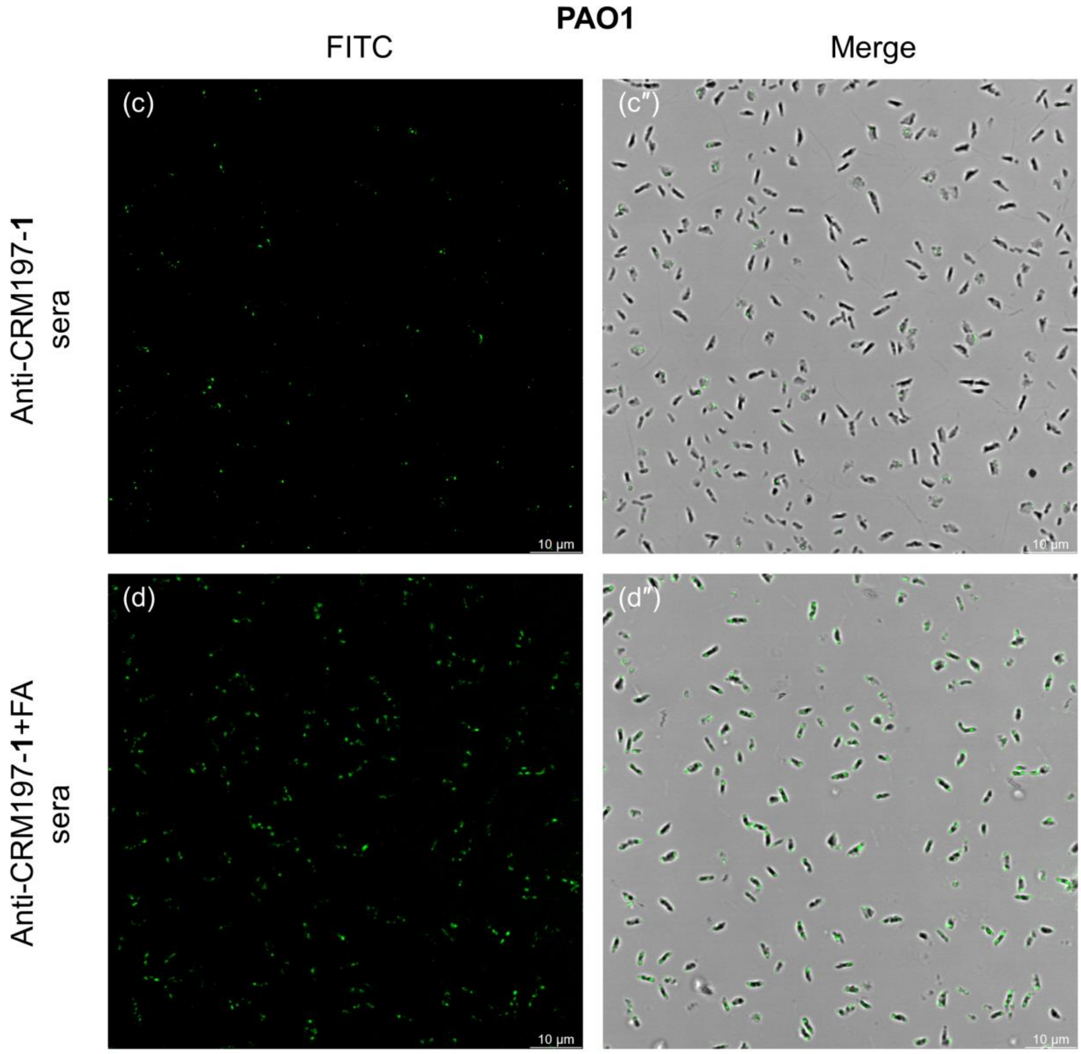

Figure S4. The enlarged images of the (c), (c"), (d), and (d") regions of Figure 3C.

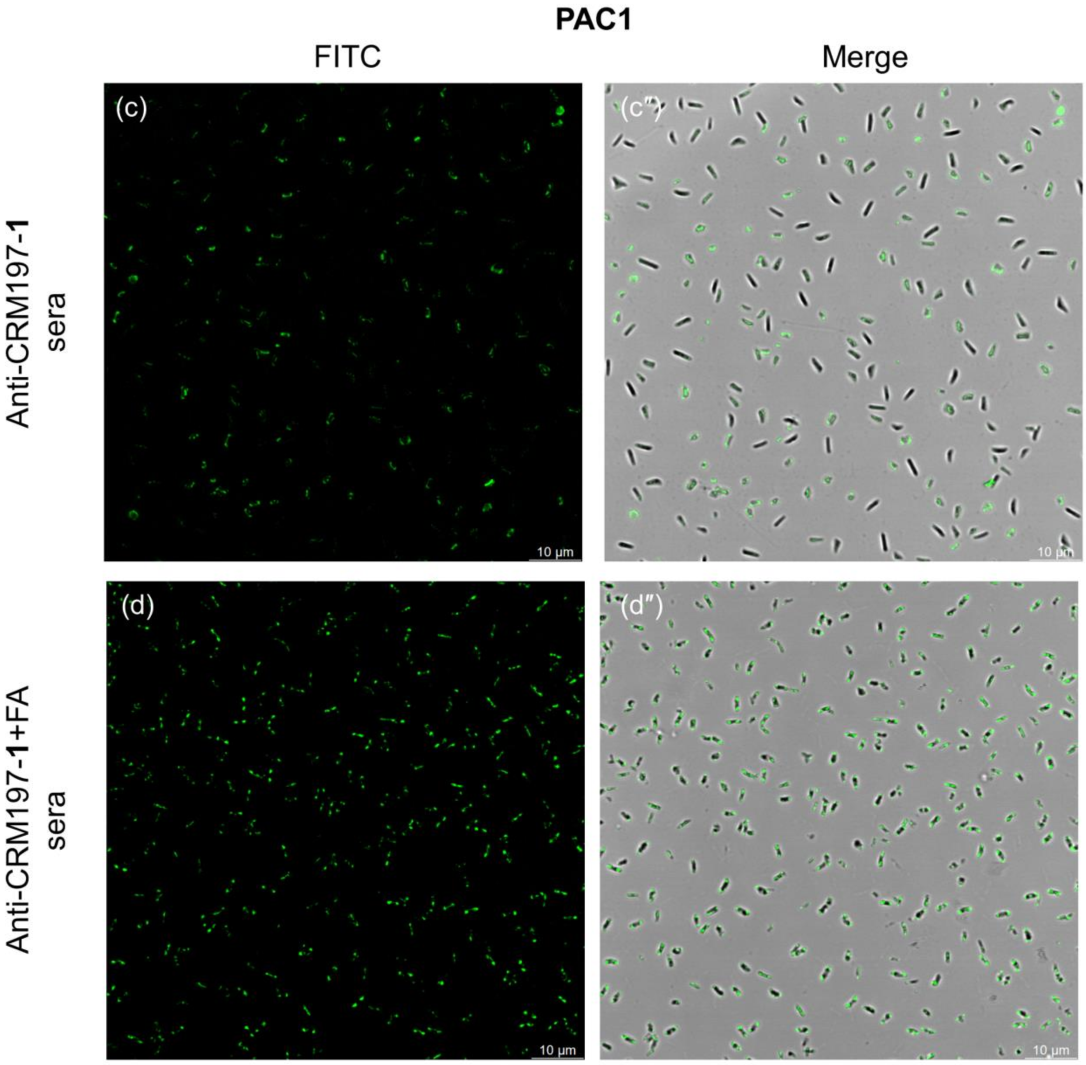

Figure S5. The enlarged images of the (c), (c"), (d), and (d") regions of Figure 3E.
